# Cold plasma-induced structural and thermal enhancements in marshmallow root mucilage-gelatin aerogels

**DOI:** 10.1016/j.crfs.2025.101027

**Published:** 2025-03-07

**Authors:** Marzieh Rownaghi, Mahdi Keramat-Jahromi, Mohammad-Taghi Golmakani, Mehrdad Niakousari

**Affiliations:** a Department of Food Science & Technology, College of Agriculture, Shiraz University, Shiraz, Iran; b Department of Mechanical Engineering of Biosystems, College of Agriculture, Shiraz University, Shiraz, Iran

**Keywords:** Aerogels, Mucilage, Nitrogen cold plasma, Rheological analysis, Structural modifications, Surface wettability

## Abstract

Aerogels are highly regarded for their low density and large surface area, attracting significant attention due to their diverse applications. This study explored nitrogen cold plasma's impact on the structure and thermal stability of mucilage-gelatin aerogels (MGA). Aerogels were prepared using marshmallow root mucilage and gelatin in a 1:1 ratio and gelatin-only as a blank under different pH conditions (5 and 7). Rheological and texture analyses identified pH 7 as optimal. Aerogels at pH 7 were then exposed to cold plasma for varying durations (0, 3, and 6 min). Thermogravimetric analysis (TGA), differential thermal analysis (DTA), and X-ray diffraction (XRD) showed enhanced thermal stability and structural changes with increased plasma exposure. Fourier-transform Infrared Spectroscopy (FTIR) revealed functional group changes, and contact angle measurements showed that 3 min of plasma treatment increased hydrophilicity (88.37–82.05°), while 6 min enhanced hydrophobicity in 1:1 MGA (93.27°). BET (Brunauer-Emmett-Teller) analyses of the MGA samples revealed changes in surface area (2.9–4.33 m^2^/g after 3 min of plasma) and BJH (Barrett-Joyner-Halenda) pore volume (0.004–0.02 cm^3^/g), with a complex trend over time. This study highlights nitrogen cold plasma's potential to enhance mucilage-based biopolymer aerogels, paving the way for advanced materials via optimized treatments.

## Highlights

• Nitrogen cold plasma enhances the thermal stability of mucilage-gelatin aerogels.
• Plasma treatment modifies structural properties, confirmed by XRD and FTIR analyses.
• Plasma treatment shifts aerogels from hydrophilic (82.05°) to hydrophobic (93.27°).
• Aerogel's surface area and pore volume increase after 3 min of nitrogen plasma exposure.
• Plasma treatment shows potential for optimizing biopolymer aerogels for diverse uses.

## Graphical abstract

**Figure:**
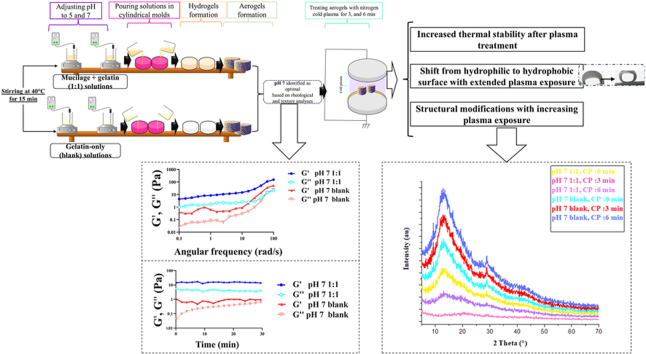

## 1 Introduction

Aerogels are exceptional solid materials characterized by a unique combination of properties often found individually in other substances. Their distinctive physical characteristics result from a porous structure and diverse chemical compositions, including organic, inorganic, or hybrid materials (S et al., 2023).

The synthesis and documentation of the first aerogel were achieved by Steven S. Kistler in 1931. Kistler produced a silica aerogel by replacing the liquid in a silica gel with a different solvent that would not disrupt the structure during drying (Kistler, 1931). This process preserved the integrity of the aerogel by converting the solvent directly to a gas at its critical temperature, avoiding evaporative loss and maintaining an intact solid network. Kistler described aerogels as "jellies" in which the original liquid is replaced by gas, resulting in minimal to no shrinkage and the retention of a solid network (Kistler, 1931).

Building on Kistler's work, modern research has broadened the scope of aerogels, with a particular focus on biopolymer-based variations. Biopolymer-based aerogels exhibit various physical, chemical, and mechanical properties, making them highly versatile for numerous applications (Halakarni et al., 2024).These aerogels are highly effective for removing organic molecules, extracting organic solvents, separating oil and water, solar-driven evaporation of water, and isolating heavy metal ions. Due to their unique characteristics, they also play a crucial role in the pharmaceutical industry (Halakarni et al., 2024).

In recent studies, significant attention has been focused on using biopolymers to create aerogels, particularly for applications in biomedicine. Aerogels made from biopolymers are being explored for various biomedical uses, including wound healing, the development of tissue engineering scaffolds, and drug delivery systems (Yahya et al., 2020). Table 1 summarizes key studies related to biopolymer-based aerogels and their applications. These findings highlight the versatility and effectiveness of biopolymer aerogels across various fields, from environmental remediation to biomedical applications (Wang et al., 2021; Ghoreishi et al., 2017; Jiang et al., 2019; Ahmadzadeh-Hashemi et al., 2023; Yang et al., 2024).

**Table 1:** Summary of key studies on biopolymer-based aerogels and their applications in various fields.

Biopolymer	Method	Application	Key findings	Reference
**Pectin**	Incorporation of PEI + EGDE cross-linker	Lead removal from water	- Maximum Pb^2+^ adsorption capacity of 373.7 mg/g at pH 5.0 - Ultralight (63.4 mg/cm^3^) - High mechanical strength (0.24 MPa at 50% strain)	Wang et al. (2021)
**Mucilage**	Supercritical carbon dioxide (SC-CO_2_) process	A promising anticancer drug loading	- Mean particle size: 82–131 nm - Drug loading efficiency: 28–52%	Ghoreishi et al. (2017)
**Gelatin**	Fabrication of gelatin/TiO_2_/PEI composite aerogel via cross-linking with glutaraldehyde	Water remediation	- Hierarchical porous structure with super amphiphilic surface - Excellent oil/water separation properties	Jiang et al. (2019)
**Gelatin and gum**	Fabrication of composite aerogels	Evaluation of gel strength and elasticity	- Gel strength and elasticity decreased with high levels of gum	Ahmadzadeh-Hashemi et al. (2023)
**Starch**	Encapsulation of Procyanidins (PC) in wheat starch aerogel (WSA)	Encapsulation of Procyanidins to enhance stability and bioactivity	- PC-WSA exhibited antioxidant activity - WSA improved the stability of PC and showed potential for practical applications	Yang et al. (2024)

Despite numerous benefits and potential uses of aerogels, they face significant challenges regarding handling and storage. The high polarity of polysaccharide gels and the open-porous nature of aerogels, especially those made from biopolymers, make them highly susceptible to moisture (Schroeter et al., 2021). This can lead to changes in their internal surfaces and potential collapse of their pores. Moreover, biopolymer aerogels, such as those made from alginate and cellulose, can experience structural disruption when exposed to polar liquids, while non-polar liquids may be absorbed into their pores. Therefore, protecting aerogels is crucial, given their sensitivity to surface conditions and storage. Hydrophobic modifications can be applied to dry biopolymer aerogels to safeguard against polar substances. This can be achieved through two main strategies: using a protective outer layer or modifying the inner porous surfaces. The appropriate method depends on the aerogel's intended application (Schroeter et al., 2021).

A solvent-free and effective method for improving aerogels' hydrophobic properties involves applying hydrophobic polymer layers through cold plasma coating. In this approach, dry aerogels are exposed to a cold plasma created by the glow discharge of gaseous hydrophobizing agents under moderate vacuum conditions (Schroeter et al., 2021; Smirnova and Gurikov, 2017). This technique has become increasingly popular due to its selective, eco-friendly, and cost-efficient nature (Schroeter et al., 2021). Cold plasma processing is particularly valued for altering the surface chemistry and the micro/nano-texture of materials (Ma et al., 2023). By generating a diverse array of surface functional groups, plasma treatment enhances the specific surface free energy, thereby modifying the wetting behavior of the aerogel. This process effectively integrates surface chemistry and texture changes, improving hydrophobicity and increasing resistance to moisture-related issues (Bormashenko et al., 2021).

In this study, marshmallow root mucilage and gelatin aerogels were prepared and exposed to cold plasma treatment. Marshmallow root mucilage was selected for its exceptional high water-holding capacity, contributing to the aerogel's overall structural integrity and functionality (Tosif et al., 2021). Gelatin was chosen for its gel-forming ability, well-documented biocompatibility, and versatility, which enhances the mechanical properties and stability of the aerogel matrix (Mushtaq et al., 2022). The novelty of this research lies in the development of a mucilage-gelatin composite aerogel and the application of cold plasma treatment to enhance its properties. While cold plasma treatment has been widely explored for modifying the characteristics of various materials, its application to aerogels made from marshmallow root mucilage—a natural, renewable biopolymer—and gelatin offers a unique and innovative approach. Unlike conventional biopolymer aerogels, which typically rely on synthetic cross-linking agents or complex fabrication processes, the mucilage-gelatin composite offers a sustainable, bio-based alternative with the potential for high mechanical strength and tunable surface properties. This study investigates the effect of cold plasma on improving the surface characteristics of these composite aerogels, particularly their hydrophobicity and structural integrity. By exploring this novel combination of cold plasma treatment with a mucilage-gelatin matrix, this research opens new avenues for optimizing the performance of biopolymer aerogels for applications in environmental remediation, biomedical engineering, and beyond.

## 2 Materials and methods

### 2.1 Materials

Marshmallow (*Althaea officinalis* L.) roots with authenticity certificates were purchased from a well-known medicinal plants dealer (Shiraz, Iran). Gelatin (type B, from bovine skin, with a bloom strength of 225) was obtained from Sigma-Aldrich (St Louis, MO, USA). Other materials were sourced from Merck (Germany) or Kimia Mavad (Tehran, Iran).

### 2.2 Methods

#### 2.2.1 Hydrogels preparation

**Fig. 1:**
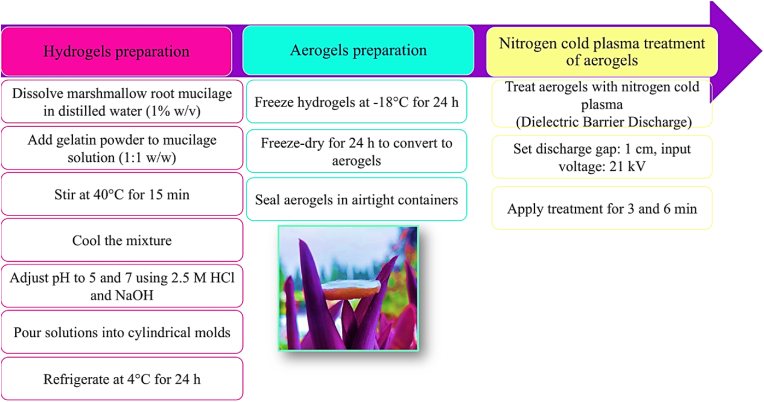
Flow chart of hydrogel and aerogel preparation and treatment process.

Hydrogels were prepared using marshmallow root mucilage, extracted via ohmic heating as described in our previous study (Rownaghi and Niakousari, 2024), and gelatin. Mucilage was dissolved in distilled water at 1% (w/v) and mixed using a magnetic stirrer (Alfa D500) for homogeneity. Gelatin was added in a 1:1 (w/w) ratio and stirred at 40 °C for 15 min. Higher mucilage concentrations did not result in gel formation. After cooling, the pH was adjusted to 5 and 7 using 2.5 M HCl and NaOH (AZ Bench Top 86502 pH meter). The mixtures were poured into cylindrical molds and refrigerated at 4 °C for 24 h to form hydrogels (Ahmadzadeh-Hashemi et al., 2023). Fig. 1 illustrates the hydrogel and aerogel preparation process, including cold plasma treatment.

#### 2.2.2 Aerogels preparation

Following hydrogel formation, the hydrogels were frozen at −18 °C for 24 h and subsequently subjected to freeze-drying (Alpha 2–4 LD plus, Christ company, Germany) for 24 h to convert them into aerogels (Ahmadzadeh-Hashemi et al., 2023; Pan et al., 2021). The resulting aerogels were sealed in airtight containers to prevent moisture absorption. A similar procedure was performed with gelatin alone as a blank control. It is worth noting that, while some studies distinguish aerogels based on the drying method (e.g., freeze-drying resulting in 'cryogels'), in the present study, the term 'aerogel' is used consistently for clarity and comparison purposes. Freeze-drying, however, generally produces cryogels with lower specific surface areas due to the formation of larger, less interconnected pores caused by ice crystal sublimation.

#### 2.2.3 Cold plasma treatment of aerogels

The aerogels were treated with nitrogen cold plasma (Dielectric Barrier Discharge) (Nikplasma Tech Co., Tehran, Iran). The discharge gap between the aluminum electrodes was set at 1 cm, and cold plasma was generated with an input voltage of 21 kV (Namjoo et al., 2022). Treatment durations of 3 and 6 min were applied to evaluate their effects on the aerogel properties. It is worth mentioning that plasma treatment was applied exclusively to the aerogels prepared at pH 7, as they exhibited better rheological properties and texture than those at pH 5.

#### 2.2.4 Determination of zeta potential

To determine the zeta potential of gelatin and mucilage, 0.5 g/L solutions of each were prepared separately in deionized water. The pH of the gelatin and mucilage solutions was adjusted to 5 and 7 using 0.1 M HCl and NaOH. After adjusting the pH, the solutions were homogenized to eliminate scattered particles and ensure uniformity. The zeta potential of each sample at the specified pH levels was then measured using a dynamic light scattering (DLS) device (SZ-100, Horiba, Japan) (Ahsan and Rao, 2017).

#### 2.2.5 Texture profile analysis

The mechanical properties of the hydrogel and aerogel samples were evaluated using the texture profile analyzer (Brookfield CT3). The samples, which were cylindrical with a diameter of 30 mm and a length of 10 mm, were analyzed. A cylindrical probe (TA4/1000, 38.1 mm diameter, 20 mm length) was used for the compression tests. The test speed was set to 1 mm/s, and each sample was compressed twice to 15% of its original height. The parameters measured included hardness, cohesiveness, and adhesiveness (Cheaburu-Yilmaz et al., 2019). All analyses were conducted in triplicate at room temperature.

#### 2.2.6 Rheological properties of hydrogels

The rheological properties of the hydrogels were analyzed using an Anton Paar MCR-302 rheometer with a cone-plate geometry (1° cone angle, 50 mm diameter). Hydrogel samples, adjusted to pH 5 and 7, were quickly transferred onto the rheometer's base plate and allowed to rest for 5 min to remove any residual shear history before testing at 25 °C. During the frequency sweep test, the storage modulus (G′) and loss modulus (G″) were evaluated over a frequency range of 0.1–100 rad/s. It is worth mentioning that an amplitude sweep test was conducted before performing the frequency sweep test to ensure that measurement was taken within the linear viscoelastic region (LVR). A time sweep test was performed, with measurements of G′ and G″ for up to 30 min, maintaining a constant shear strain of 1% and a frequency of 1 Hz (Cheaburu-Yilmaz et al., 2019; Ng et al., 2021).

#### 2.2.7 Wettability measurements of aerogels

The wettability of the aerogels was assessed using the Jican-CAG20 contact angle meter. A droplet of 2 μL of water was dripped onto the surface of each sample. The contact angle, which is the angle between the water droplet and the sample's surface, was measured using ImageJ software (version 1.52v) for accurate calculation (Zhu et al., 2019).

#### 2.2.8 Surface and pore characterization (BET, Langmuir, and BJH) of aerogels

The aerogels' specific surface area (BET and Langmuir), total pore volume, and mean pore diameter were analyzed using nitrogen adsorption-desorption isotherms at −196 °C (BELSORP MAX G). Additionally, Barrett-Joyner-Halenda (BJH) methods were employed to determine the pore volume, peak pore radius, and specific surface area. Before analysis, the aerogels, each weighing between 0.15 and 0.30 g, were cut into smaller fragments and degassed under a vacuum at 115 °C for 4 h (Ubeyitogullari and Ciftci, 2020).

#### 2.2.9 Aerogels morphology

The morphology of the aerogels was investigated using scanning electron microscopy (SEM, TESCAN-Vega3). The samples were coated with an ultrathin layer of gold using a sputter coater (Q150R-ES). SEM images were captured at a magnification of 70 × .

#### 2.2.10 XRD pattern of aerogels

The crystalline structure of the aerogels was analyzed using X-ray diffraction (XRD-D8 ADVANCE, Bruker, Germany). The diffraction patterns were obtained at 40 kV and 20 mA. Measurements were conducted over a 2θ range of 5–70°, employing CuKα radiation with a wavelength of 0.154 nm, and were scanned at a rate of 1°/min (Suttiruengwong et al., 2024). The obtained diffraction patterns were analyzed using Origin Pro 2018 software to determine peak positions and Full Width at Half Maximum (FWHM) values.

#### 2.2.11 FT-IR spectroscopy of aerogels

FT-IR spectroscopy was utilized to analyze the functional groups present in the aerogels. The measurements were carried out using a Bruker Tensor II spectrometer, with spectral data collected from 400 to 4000 cm^−1^. A total of 45 scans were performed at a resolution of 4 cm^−1^ (Pantić et al., 2024).

#### 2.2.12 Thermal behavior of aerogels

Thermogravimetric analysis (TGA), derivative thermogravimetric (DTG), and differential thermal analysis (DTA) were performed using a Thermogravimetric Analyzer (TA Instruments Q50). The analysis was conducted at a scan rate of 10 °C/min, covering a temperature range from 25 to 700 °C, under an argon atmosphere (Vasil'kov et al., 2021).

### 2.3 Statistical analysis

Analyses were carried out in triplicates of each setup. Results are expressed as Mean ± STD. Statistical significant difference (p < 0.05) was determined using Duncan's multiple range test after analyzing the variance (ANOVA) in a completely randomized design using SAS version 9.4.

## 3 Results and discussion

### 3.1 Zeta potential determination

**Fig. 2:**
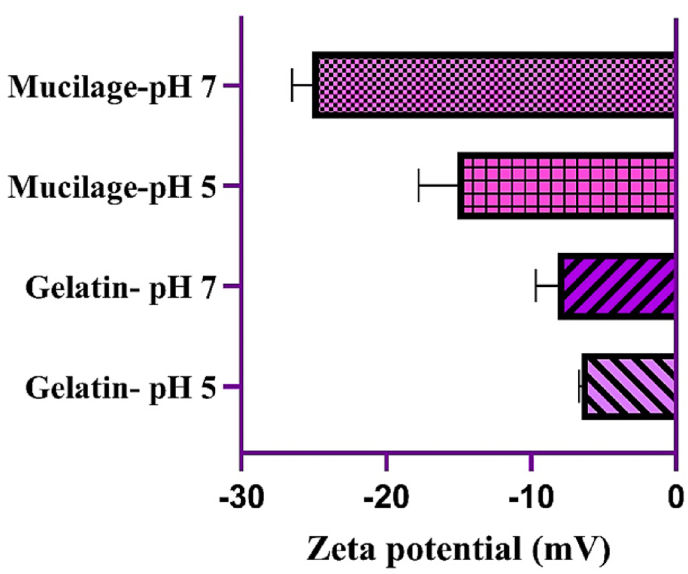
Zeta potential values of gelatin and mucilage in pH 5 and 7.

The zeta potential of gelatin and mucilage was measured at two pH levels (5 and 7) to assess surface charge characteristics (Fig. 2). At pH 5, gelatin type B exhibited a zeta potential of −6.53 ± 0.12 mV, which became slightly more negative (−8.17 ± 1.21 mV) at pH 7. In comparison, mucilage demonstrated a significantly more negative zeta potential, ranging from −15.07 ± 2.20 mV at pH 5 to −25.10 ± 1.14 mV at pH 7. The pronounced shift in mucilage's zeta potential with increasing pH underscores its higher sensitivity to pH variations than gelatin.

The markedly negative zeta potential of mucilage suggests a higher density of ionizable functional groups, such as carboxyl or sulfate groups, contributing to enhanced electrostatic repulsion and stability against aggregation. These properties are consistent with prior findings, where polymers with high surface charge densities were shown to resist aggregation effectively (Ahsan and Rao, 2017; Souza et al., 2021).

Zeta potential measurements have been widely applied to study biopolymer interactions. For instance, studies involving gum arabic reported zeta potentials of approximately −25 mV at pH 5 and -27 mV at pH 7 (Badke et al., 2019). Similarly, Ahsan and Rao (2017) observed comparable zeta potential values for gelatin type B (Ahsan and Rao, 2017). Such findings align with the results obtained in this study.

Despite the similarity in negative charges between gelatin and mucilage, their interactions can be influenced by structural and functional group differences. Gelatin's amino (-NH2) and carboxyl (-COOH) groups facilitate cross-linking through hydrogen bonding and ionic interactions, potentially interacting with mucilage's hydroxyl (-OH) groups. These interactions contribute to gel network formation, which can be further modulated by pH, temperature, and charge density variations (Sarika et al., 2015; Wanasingha et al., 2021).

While previous studies have explored hydrogel formation using biopolymers with opposing charges, the potential for gelation between polymers of similar charge densities remains underexplored. For example, Ahmadzadeh-Hashemi et al. (2023) demonstrated hydrogel formation via electrostatic attraction between negatively charged quince seed gum (−18 mV) and positively charged gelatin (+13 mV) (Ahmadzadeh-Hashemi et al., 2023). Similarly, Zhang et al. (2019) reported hydrogel formation at pH 5.5 using gelatin (near 0 mV) and highly negative Tara gum (−40 mV) (Zhang et al., 2019a). These studies emphasize the role of electrostatic interactions in hydrogel stabilization.

In the case of mucilage and gelatin, cooperative gelation mechanisms may dominate, driven by their complementary functional groups rather than their electrostatic interactions alone. Such insights highlight the complex interplay of molecular structure, environmental factors, and interaction dynamics in the design of composite hydrogels (Beaumont et al., 2021; Li et al., 2021).

### 3.2 Texture profile analysis

The mechanical properties of hydrogel and aerogel samples were assessed via texture profile analysis, focusing on hardness, adhesiveness, and cohesiveness (Table 2). The pH 7 1:1 hydrogel, a composite of mucilage and gelatin, exhibited the highest hardness (19.15 ± 0.57 g), significantly higher than other samples (p < 0.05), indicating that mucilage enhances the mechanical strength of gelatin-based hydrogels. Similar improvements in mechanical properties have been noted in studies integrating non-fouling polymers and antibacterial polysaccharides, such as chitosan, in double-network hydrogels (Zhang et al., 2019b). The superior hardness at neutral pH likely results from the optimal ionization of gelatin compared to its behavior at its isoelectric point (Ahsan and Rao, 2017).

**Table 2:** Mechanical properties of hydrogel and aerogel samples.

Sample	Type	Hardness (g)	Adhesiveness (mJ)	Cohesiveness
**Hydrogels**				
pH 7 1:1	Hydrogel	19.15 ± 0.57^a^∗	0.05 ± 0.01^b^	1.07 ± 0.14^a^
pH 7 blank	Hydrogel	17.50 ± 0.50^b^	0.14 ± 0.01^a^	0.83 ± 0.05^b^
pH 5 1:1	Hydrogel	16.42 ± 1.38^b^	0.06 ± 0.01^b^	0.84 ± 0.06^b^
pH 5 blank	Hydrogel	16.39 ± 0.19^b^	0.15 ± 0.04^a^	0.74 ± 0.11^b^
**Aerogels**				
pH 7 1:1	Aerogel	426.20 ± 1.49^a^	0.02 ± 0.00^a^	0.79 ± 0.02^b^
pH 7 blank	Aerogel	43.11 ± 0.78^c^	0.02 ± 0.00^a^	0.81 ± 0.03^b^
pH 5 1:1	Aerogel	402.90 ± 0.26^b^	0.01 ± 0.00^b^	0.61 ± 0.02^c^
pH 5 blank	Aerogel	24.39 ± 0.23^d^	0.01 ± 0.00^b^	1.06 ± 0.00^a^

∗Different letters within a column represent significant differences at p < 0.05. Statistical analyses for hydrogels and aerogels were conducted separately. pH 7 1:1: Hydrogel/aerogel composed of mucilage and gelatin in a 1:1 ratio at pH 7. pH 7 blank: Hydrogel/aerogel composed of gelatin only at pH 7. pH 5 1:1: Hydrogel/aerogel composed of mucilage and gelatin in a 1:1 ratio at pH 5. pH 5 blank: Hydrogel/aerogel composed of gelatin only at pH 5.

For adhesiveness, the pH 7 and 5 1:1 hydrogels showed the lowest values (0.05 and 0.06 mJ), demonstrating mucilage's role in reducing stickiness. Previous studies on mucilage's impact on hydrogel mechanical properties support these findings (Chokboribal et al., 2023). For cohesiveness, the pH 7 1:1 hydrogel exhibited the highest value (1.07 ± 0.14), showing superior structural integrity compared to the pH 5 formulations and the gelatin-only controls. This trend contrasts with a study on a 50:50 κ-carrageenan/quince seed mucilage hydrogel, where higher hardness correlated with lower cohesiveness (Forghani and Almasi, 2024).

For aerogels, the pH 7 1:1 aerogel demonstrated exceptional hardness (426.20 ± 1.49 g), consistent with the hydrogel form, suggesting the freeze-drying process preserved the structural network formed at neutral pH (Tannert et al., 2015). The pH 5 1:1 aerogel showed the lowest adhesiveness (0.01 mJ), similar to the pH 5 blank aerogel, indicating minimal stickiness. The lower zeta potential at pH 5 could contribute to the reduced adhesiveness by lowering electrostatic repulsion (Moreno Baqueiro Sansao et al., 2021). In contrast, the higher zeta potential at pH 7 slightly increased adhesiveness in the pH 7 1:1 aerogel. The differences in trends between hydrogel and aerogel forms highlight the impact of freeze-drying on surface interactions and adhesion (Ghafar, 2018). Regarding cohesiveness, the pH 5 1:1 aerogel showed the lowest value (0.61 ± 0.02), suggesting reduced structural integrity compared to other samples. The pH 5 blank aerogel, in contrast, exhibited the highest cohesiveness. This could be due to the stable gelatin network at pH 5, which is disrupted in the pH 5 1:1 aerogel by mucilage, which introduces complexity to the gel matrix. The freeze-drying process may also better preserve the gelatin-only aerogel, contributing to its higher cohesiveness (Borella et al., 2023).

Overall, the pH 7 formulations (both hydrogel and aerogel) generally exhibited better mechanical properties, particularly in terms of hardness and cohesiveness, compared to the pH 5 samples, suggesting the neutral pH provides more favorable conditions for the interactions between mucilage and gelatin.

### 3.3 Rheological properties of hydrogels

**Fig. 3:**
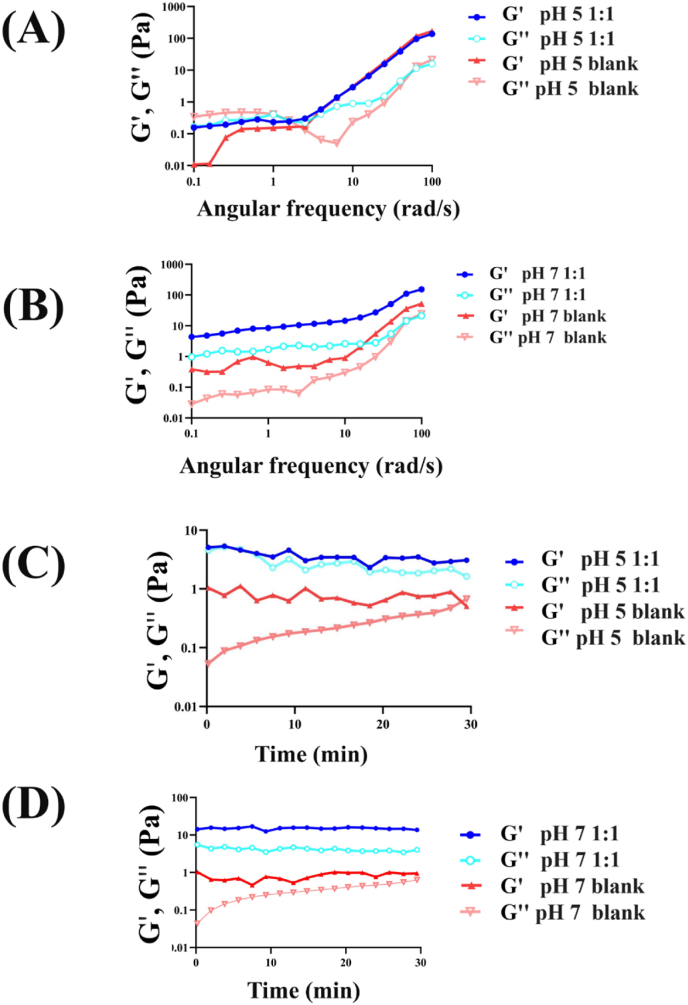
Rheological properties of hydrogels at different pH levels. A: Frequency sweep rheological properties of pH 5 (1:1 and blank). B: Frequency sweep rheological properties of pH 7 (1:1 and blank). C: Time sweep rheological properties of pH 5 (1:1 and blank). D: Time sweep rheological properties of pH 7 (1:1 and blank). 1:1: Hydrogel composed of mucilage and gelatin in a 1:1 ratio. blank: Hydrogel composed of gelatin only.

Fig. 3A and B compared the frequency sweep rheological properties of hydrogels at pH 5 and pH 7, including 1:1 and blank formulations, using the storage modulus (G′) and loss modulus (G″) across 0.1–100 rad/s. Fig. 3A showed that at pH 5, both 1:1 and blank hydrogels exhibited increases in G′ and G″ with frequency. The 1:1 hydrogel consistently showed higher G′ values than the blank, while the blank displayed higher G″ at lower frequencies, surpassing the 1:1 at higher frequencies, indicating a crossover point. Fig. 3B revealed that at pH 7, both hydrogels exhibited higher G′ than G″, indicating stronger elasticity. The pH 7 1:1 hydrogel demonstrated higher G′ and G″ values compared to the blank across all frequencies.

Overall, the pH 7 1:1 hydrogel demonstrated superior rheological properties compared to the pH 5 1:1, with higher G′ and G″ values, reflecting enhanced elasticity and damping. In contrast, the pH 5 blank exhibited a more variable damping response, while the pH 7 blank displayed a more stable profile. These findings align with Afzal et al. (2018), who reported increased elasticity in composite hydrogels at neutral pH (Afzal et al., 2018). Similarly, Jongprasitkul et al. (2024) found that neutral pH conditions enhanced the mechanical strength and stability of hydrogels, while acidic conditions weakened the network (Jongprasitkul et al., 2024). The superior mechanical properties of the pH 7 1:1 hydrogel further support these observations. This comprehensive analysis demonstrates that pH 7 1:1 hydrogel offers the best rheological and mechanical performance among the tested hydrogels, aligning with the expected effects of pH on hydrogel network stability and strength (Wang et al., 2023).

Fig. 3C and D presented the time sweep rheological properties of pH 5 and pH 7 hydrogels over 30 min. Fig. 3C showed that for pH 5, the storage modulus (G′) consistently exceeded the loss modulus (G″), indicating predominant elasticity. The pH 5 1:1 hydrogel exhibited higher G′ and G″ values than the blank, reflecting better elasticity and structural integrity. Fig. 3D revealed that for pH 7, G′ remained higher than G″ throughout the observation period, with the pH 7 1:1 hydrogel showing higher G′ and G″ values than the blank.

When comparing pH conditions, the pH 7 1:1 hydrogel showed higher G′ and G″ values than its pH 5 counterpart. Similarly, Bertasa et al. (2020) observed that agar hydrogels showed high stability, with minimal changes in G′ and G″ over time (Bertasa et al., 2020), while Ahmadzadeh-Hashemi et al. (2023) found that quince seed gum and gelatin hydrogels exhibited higher G′ than G″, although gels with a 1:1 ratio showed lower moduli than pure gelatin gels (Ahmadzadeh-Hashemi et al., 2023).

Time sweep rheology is used to observe structural changes in materials over time, including solvent evaporation, curing, gelation, polymer degradation, or recovery (Stojkov et al., 2021). This technique monitors the impact of these changes on the material's rheological properties. The gelation time is marked by the crossover of the storage modulus (G′) and loss modulus (G″), indicating the gelation kinetics (Stojkov et al., 2021). Additionally, monitoring these changes helps assess the stability of hydrogels.

In addition to the observed time sweep rheological properties, the pH level influences the interaction between gelatin and mucilage. Despite gelatin and mucilage having similar charges, the crosslinking between them is more effective at pH 7 than pH 5. This is likely due to pH 7 being further from the isoelectric point of gelatin (pH 4.7), where gelatin's charge interactions are less neutralized and thus more conducive to forming crosslinks (Ji et al., 2022). This improvement can be attributed to the more effective crosslinking network at neutral pH, which supports stronger gel formation and better time sweep rheological properties.

### 3.4 Wettability measurements of aerogels

**Fig. 4:**
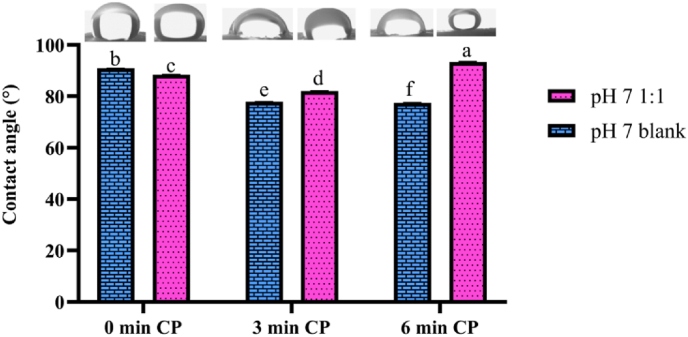
Contact angle measurements of pH 7 1:1 and blank aerogels exposed to cold plasma for varying durations. 1:1: Aerogel composed of mucilage and gelatin in a 1:1 ratio. blank: Aerogel composed of gelatin only. Different letters represent significant differences at p < 0.05.

The cold plasma was conducted on the aerogels at pH 7, which demonstrated superior rheological in hydrogel form and mechanical properties in both hydrogel and aerogel form. Contact angle measurements revealed significant differences (p < 0.05) in response to varying plasma exposure times (0, 3, and 6 min) for both pH 7 1:1 (mucilage-gelatin) and pH 7 blank (gelatin-only) aerogels (Fig. 4). For the pH 7 1:1 aerogels, the contact angle decreased from 88.37 ± 0.12° at 0 min to 82.05 ± 0.08° after 3 min, then increased to 93.27 ± 0.26° after 6 min. This increase suggests a complex interaction between cold plasma treatment and the gelatin-mucilage mixture. The initial decrease in contact angle likely results from the formation of surface polar groups or changes in surface roughness (Gojda et al., 2022), while the subsequent increase may reflect changes in surface composition or morphology, such as the reformation of hydrophobic domains or increased surface roughness, which reduce wettability over time (Neves et al., 2024).

For the pH 7 blank aerogel, the contact angle decreased consistently from 90.83 ± 0.08° at 0 min to 77.88 ± 0.06° at 3 min and slightly further to 77.50 ± 0.02° at 6 min. This decrease indicates enhanced surface hydrophilicity, likely due to the introduction of polar functional groups or surface etchings that increase surface energy, making the surface more receptive to water (Bertin et al., 2024). Although this study is pioneering in applying cold plasma treatment to MGA, existing research provides valuable context. Previous studies have extensively investigated the effects of plasma treatment on various surfaces, such as polymers and metals, where plasma exposure has been shown to modify surface energy and wettability by introducing polar functional groups or altering surface roughness (Schroeter et al., 2021; Cheng et al., 2012; Goiana et al., 2022; He et al., 2021; Mandolfino et al., 2014). Cheng et al. (2012) reported that plasma treatment of sodium alginate aerogels increased hydrophobicity up to 20 min of exposure (Cheng et al., 2012). Similarly, Goiana et al. (2022) observed increased hydrophobicity in various films, including gelatin, with extended plasma treatment (Goiana et al., 2022). Additionally, He et al. (2021) demonstrated that cold plasma treatment significantly increased the hydrophobicity of carboxymethyl chitosan aerogels, achieving a contact angle of 117° after 20 min of exposure at 60 W (He et al., 2021). In contrast, Kang et al. (2014) focused on generating hydrophilic groups, specifically nitrogen oxides, on polyimide films, which substantially reduced the water contact angle to below 30° (Kang et al., 2014).

The differences observed in our study highlight the impact of material composition on plasma treatment outcomes. While gelatin-based aerogels showed consistent hydrophilicity, the mucilage-gelatin mixture exhibited a more complex behavior, initially increasing in hydrophilicity before returning to hydrophobicity. This suggests that the interactions between plasma treatment and the aerogel matrix are critical in tailoring material properties. Future studies should investigate these interactions in other aerogels and under varying plasma conditions. Additionally, exploring different plasma gases, treatment durations, and power levels could provide further insights into the broader effects of plasma treatment on aerogel properties.

### 3.5 Surface, and pore characterization (BET, Langmuir, and BJH) of aerogels

The effects of cold plasma (CP) exposure on surface and pore properties of two aerogel types (pH 7 1:1 and pH 7 blank) were evaluated at 0, 3, and 6 min (Table 3). Parameters measured include specific surface area, total pore volume, mean pore diameter (BET), specific surface area (Langmuir), pore volume, pore radius at peak, and specific surface area (BJH). Notably, while BET and Langmuir analyses provide total surface area, BJH analysis focuses on mesopore size distribution and volume. Combining these methods ensures a comprehensive characterization of the aerogel's porous structure.

**Table 3:** Impact of cold plasma exposure time on surface and pore properties of aerogels.

Sample	CP exposure time	BET			Langmuir	BJH		
		Specific surface area (m^2^/g)	Total pore volume (cm^3^/g)	Mean pore diameter (nm)	Specific surface area (m^2^/g)	Pore volume (cm^3^/g)	Pore radius at peak (nm)	Specific surface area (m^2^/g)
pH 7 1:1	0 min	2.90	0.004	6.22	5.78	0.004	1.85	3.22
	3 min	4.33	0.010	12.12	12.78	0.020	1.85	15.19
	6 min	3.12	0.004	5.43	9.99	0.003	1.64	2.27
pH 7 blank	0 min	7.67	0.010	4.31	32.44	0.010	1.64	8.26
	3 min	1.28	0.002	8.64	1.74	0.002	1.64	1.44
	6 min	6.78	0.010	4.70	6.57	0.010	1.21	9.19

1:1:Aerogel composed of mucilage and gelatin in a 1:1 ratio. blank: Aerogel composed of gelatin only.

For pH 7 1:1 aerogels, cold plasma exposure initially enhanced surface area and pore volume, followed by a complex trend. At 0 min, BET-specific surface area was 2.90 m^2^/g, total pore volume 0.004 cm^3^/g, and mean pore diameter 6.22 nm. After 3 min, surface area rose to 4.33 m^2^/g, pore volume to 0.01 cm^3^/g, and pore diameter to 12.12 nm, indicating improved porosity. By 6 min, the surface area dropped to 3.12 m^2^/g, pore volume to 0.004 cm^3^/g, and pore diameter to 5.43 nm.

This indicates that while there was an initial increase in surface area and pore volume with cold plasma exposure, prolonged exposure led to a decrease, possibly due to surface restructuring or loss of active sites (Basak and Annapure, 2022). This observation aligns with the contact angle measurements from the previous section, where the contact angle of pH 7 1:1 aerogels initially decreased after 3 min of exposure, reflecting increased hydrophilicity and enhanced water affinity due to surface activation. However, after 6 min, the contact angle increased, indicating a return to more hydrophobic characteristics. This trend is consistent with the decrease in surface area and pore volume observed at longer exposure times, likely resulting from surface restructuring or the formation of hydrophobic domains. Thus, while initial plasma treatment improves wettability by expanding surface area and pore volume, extended exposure can reduce hydrophilicity as the surface undergoes changes that decrease water interaction.

The pH 7 1:1 aerogels showed a similar trend in Langmuir and BJH measurements. Langmuir-specific surface area increased from 5.78 m^2^/g at 0 min to 12.78 m^2^/g after 3 min, then decreased to 9.99 m^2^/g at 6 min. BJH measurements revealed an increase in specific surface area and pore volume after 3 min (specific surface area of 15.19 m^2^/g, pore volume of 0.02 cm^3^/g), but these values decreased to 2.27 m^2^/g and 0.003 cm^3^/g, respectively, by 6 min. This suggests that plasma exposure initially enhances surface properties, but extended treatment may reduce these benefits.

For the pH 7 blank aerogels, the effects of cold plasma exposure were initially more pronounced. At 0 min, the BET-specific surface area was 7.67 m^2^/g, with a total pore volume of 0.01 cm^3^/g and a mean pore diameter of 4.31 nm. After 3 min, the BET-specific surface area dropped to 1.28 m^2^/g, pore volume to 0.002 cm^3^/g, and mean pore diameter increased to 8.64 nm, indicating reduced surface area and pore volume. Langmuir and BJH measurements showed similar trends, with Langmuir surface area dropping to 1.74 m^2^/g and BJH to 1.44 m^2^/g.

By 6 min of exposure, BET-specific surface area increased to 6.78 m^2^/g, total pore volume to 0.01 cm^3^/g, and mean pore diameter decreased to 4.70 nm. Langmuir and BJH measurements also reflected this recovery, with specific surface areas of 6.57 m^2^/g and 9.19 m^2^/g, respectively. This rebound suggests a reactivation or restructuring of the aerogel. This trend is consistent with contact angle measurements, which showed slower decreases over time, indicating stabilization of wettability. Extended exposure may result in a balanced surface structure, influencing both surface area and wettability.

Research by Bulbul et al. (2019) on xanthan gum demonstrated that plasma treatment can influence surface and pore characteristics. BET analysis of xanthan gum treated with cold plasma at different power levels and durations showed that higher power and longer treatment times resulted in larger surface areas and altered pore structures. The most intense treatment (60 W for 20 min) achieved the highest surface area, 46.09 m^2^/g, while treatment at 50 W for 15 min resulted in the largest average pore diameter and total pore volume (Bulbul et al., 2019).

Similarly, the impact of cold plasma on bentonite clay revealed subtle changes in surface properties. Raw bentonite had a surface area of 64.2 m^2^/g and a pore volume of 0.0923 cm^3^/g, while plasma-treated bentonite showed a slight increase in surface area to 65.3 m^2^/g and a comparable pore volume of 0.0929 cm^3^/g. This modest increase indicates that cold plasma treatment has less pronounced effects on surface area compared to other methods (Şahin et al., 2015).

Studies on other porous materials, such as alginate and cellulose aerogels, further demonstrate plasma treatment's influence. In alginate aerogels, BJH desorption analysis showed that smaller pores (3–7 nm) disappeared after 5 min of plasma treatment, replaced by new mesopores (15–25 nm). For cellulose aerogels, plasma treatment created new pores in the same size range, expanding the pore distribution and increasing total pore volume in the lower pore radius range (Schroeter et al., 2021).

These modifications have practical implications, enhancing material properties for catalysis, adsorption, or environmental remediation applications (Rao et al., 2023). Future research should investigate the long-term stability of plasma-treated materials and optimize treatment parameters for specific applications to fill gaps in our understanding of plasma treatment effects.

### 3.6 Aerogels morphology

**Fig. 5:**
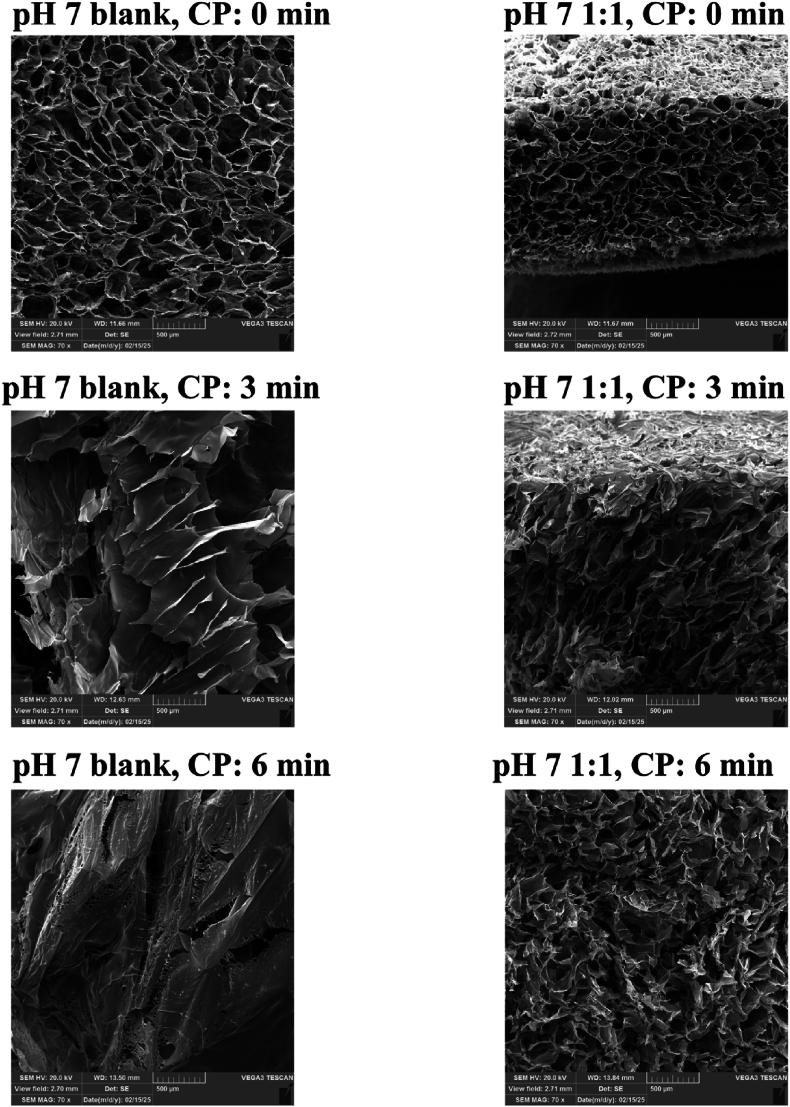
Morphology of aerogels subjected to cold plasma (CP) exposure for 0, 3, and 6 min. 1:1: Aerogel composed of mucilage and gelatin in a 1:1 ratio. blank: Aerogel composed of gelatin only. All the aerogels prepared at pH 7.

The SEM images (Fig. 5) revealed significant structural differences between the pH 7 blank and pH 7 1:1 aerogels at different cold plasma (CP) exposure times. At 0 min, both aerogels displayed a porous and interconnected network. However, the pH 7 1:1 aerogel exhibited slightly smaller and denser pores compared to the blank aerogel, which had a more open and irregular structure. This observation aligns with the BET data, which showed a higher surface area for the blank aerogel, indicating greater overall porosity.

After 3 min of CP treatment, the blank sample showed a transition towards a more sheet-like structure with partially collapsed pores, suggesting densification due to plasma-induced restructuring. This structural change corresponds with the decrease in surface area from 7.67 m^2^/g to 1.28 m^2^/g and the reduction in pore volume observed in the BET analysis. In contrast, the pH 7 1:1 aerogel retained more of its porous architecture but exhibited signs of compaction, aligning with the increase in surface area.

At 6 min, the blank aerogel displayed elongated, fused structures with fewer visible pores, indicating substantial collapse and fusion of the porous network. Conversely, the pH 7 1:1 aerogel still maintained some level of porosity, though evident deformation was observed. This suggests that prolonged plasma exposure may lead to excessive structural modification, reducing the material's surface area and pore integrity. Interestingly, The BET results for the blank aerogel showed improved surface area and pore volume at this stage, likely due to the reorganization of fused structures, which may lead to the formation of new surface features. These findings indicate that cold plasma treatment significantly modifies the structure of the aerogel, potentially enhancing its surface properties for specific applications such as drug delivery or filtration.

### 3.7 XRD pattern of aerogels

**Fig. 6:**
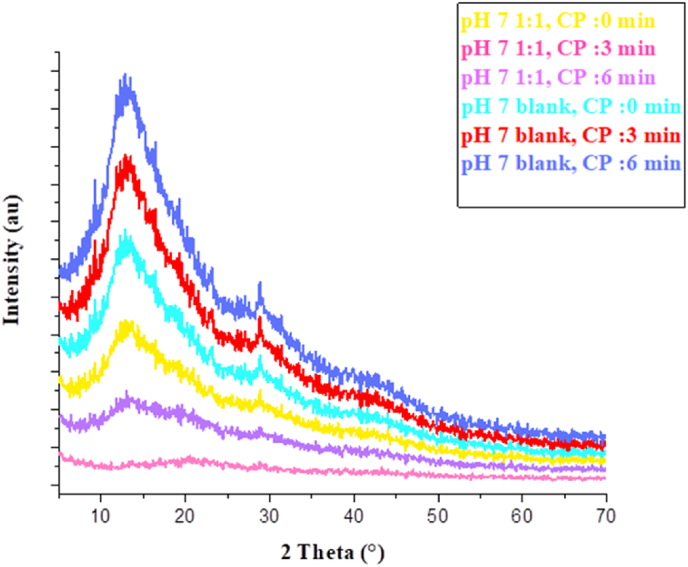
X-ray diffraction (XRD) patterns of aerogels subjected to varying cold plasma (CP) exposure times. 1:1: Aerogel composed of mucilage and gelatin in a 1:1 ratio. blank: Aerogel composed of gelatin only. All the aerogels prepared at pH 7.

X-ray diffraction (XRD) analysis (Fig. 6) revealed structural changes in aerogels induced by plasma treatment, indicating a transition towards an amorphous structure.

For the pH 7 blank, the peak position shifted slightly from 13.54° at 0 min to 13.48° after 3 min, and 13.52° after 6 min, suggesting minor variations in lattice spacing with increased plasma exposure. These shifts indicated subtle adjustments in the gelatin component's lattice spacing while maintaining overall crystalline stability. The Full Width at Half Maximum (FWHM) increased from 7.13° at 0 min to 7.51° after 3 min and 7.82° after 6 min, reflecting greater peak broadening. This broadening signifies increasing structural disorder or reduced crystallite size (Lakshmi et al., 2015; Zhang et al., 2015) as a result of cumulative plasma effects. These observations suggest slight structural modifications while preserving a generally stable crystalline arrangement.

In contrast, at pH 7 with a 1:1 ratio, plasma treatment induced notable structural changes. The peak position shifted significantly from 13.59° to 21.48° after 3 min, suggesting a phase transition or strong interactions between mucilage and gelatin. By 6 min, partial recovery was observed as the peak returned to 13.99°. The FWHM increased from 6.98° to 7.45° and 7.62°, indicating heightened disorder. These findings demonstrate pronounced structural transformations in the pH 7 1:1 aerogel under plasma treatment, marked by significant peak shifts and broadening, contrasting with the milder changes in the gelatin control.

The effects of cold plasma treatment on crystalline materials have been extensively documented. Plasma exposure induces structural changes, often reflected in peak position shifts and FWHM increases. For example, Atta and El-Sayed (2015) reported broader diffraction peaks and reduced crystallinity in polyimide films, attributing these changes to smaller crystallite sizes and increased disorder (Atta and El-Sayed, 2015). Similarly, Otálora González et al. (2024) observed reduced crystallinity in cassava starch, with the degree of crystallinity decreasing from 29% to 22% due to reactive plasma species disrupting ordered structures (Otálora González et al., 2024). However, Bie et al. (2016) noted that despite oxygen or helium plasma treatment, cassava starch maintained its A-type polymorphic structure, with main peaks at ∼15°, 17°, 18°, and 23° (2θ), indicating that the crystalline phase can remain unchanged under certain conditions (Bie et al., 2016).

These alterations in crystallinity and peak broadening have practical implications. Decreased crystallinity and increased disorder can influence thermal stability, optical properties, adhesion, and barrier characteristics in coatings or films (Primc and Mozetič, 2024). For biomedical and catalytic applications, such changes may affect biocompatibility, degradation, or catalytic efficiency (Bal et al., 2018). Future studies should aim to optimize plasma parameters to balance material properties, evaluate long-term stability, and compare treatment effects across diverse materials.

### 3.8 FT-IR spectroscopy of aerogels

**Fig. 7:**
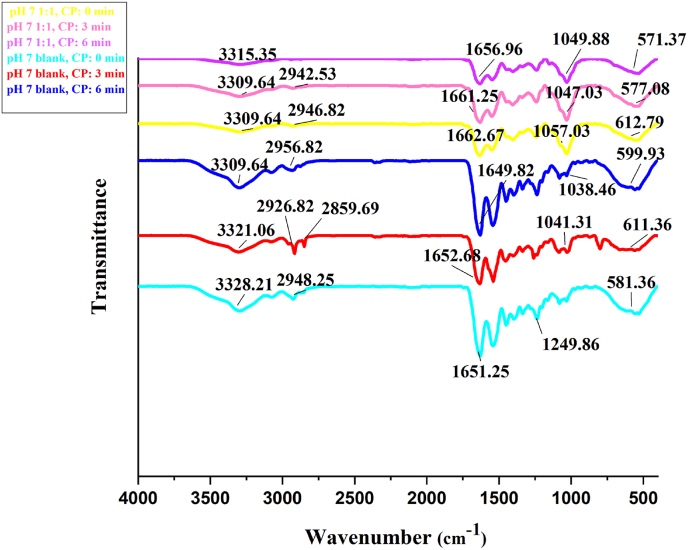
FT-IR spectra of aerogels subjected to varying cold plasma (CP) exposure times. 1:1: Aerogel composed of mucilage and gelatin in a 1:1 ratio. blank: Aerogel composed of gelatin only. All the aerogels prepared in pH 7.

The FTIR analysis revealed the impact of cold plasma treatment on aerogel molecular structures (Fig. 7). In blank (gelatin-only, pH 7) aerogels, the N-H stretching vibration peak (Tian et al., 2022) shifted from 3328.21 cm^−1^ (0 min) to 3321.06 cm^−1^ (3 min) and 3309.64 cm^−1^ (6 min), indicating progressive plasma-induced alterations in N-H bonds, likely due to radicals (Sharath Kumar et al., 2024).

For mucilage-gelatin aerogels (1:1, pH 7), the peak remained stable at 3309.64 cm^−1^ for 0–3 min but shifted to 3315.35 cm^−1^ at 6 min. Initial peak alignment with gelatin-only samples suggested mucilage had minimal influence on N-H stretching pre-treatment. However, the 6-min shift highlighted distinct structural changes in the mucilage-gelatin matrix under prolonged plasma exposure.

Furthermore, For the pH 7 blank (gelatin-only) aerogels, the C-H stretching vibration peak, initially at 2948.25 cm^−1^ for 0 min of plasma exposure, shifted to 2926.82 cm^−1^ after 3 min and further to 2956.82 cm^−1^ after 6 min. These shifts, associated with C-H stretching vibrations (Bazylewski et al., 2017), suggest that plasma treatment modifies the aliphatic C-H bonds in gelatin, likely due to plasma-induced oxidation or other changes.

For the pH 7 1:1 (mucilage-gelatin 1:1 ratio) aerogels, the peak was at 2946.82 cm^−1^ for 0 min and shifted to 2942.53 cm^−1^ after 3 min. Notably, no peak appeared in this region after 6 min of plasma treatment. This absence of a peak suggests that extended plasma exposure resulted in significant degradation or alteration of the C-H bonds in the mucilage-gelatin matrix. Additionally, the pH 7 blank aerogels showed a peak at 2859.69 cm^−1^ after 3 min of plasma exposure, indicating modifications in the aliphatic C-H bonds of gelatin. This suggests that while some C-H bonds were affected during this time, not all were modified by the plasma treatment.

Further FTIR analysis showed significant shifts in the amide I band, corresponding to the C=O stretching vibration (Usoltsev et al., 2019). For the pH 7 blank (gelatin-only) aerogels, the peak at 1651.25 cm^−1^ for 0 min of plasma exposure shifted slightly to 1652.68 cm^−1^ after 3 min and to 1649.82 cm^−1^ after 6 min. These minor shifts suggest that cold plasma treatment caused slight changes in the carbonyl group environment in the gelatin matrix, likely due to plasma-induced chemical modifications.

In contrast, the mucilage-gelatin 1:1 aerogel initially peaked at 1662.67 cm^−1^ for 0 min, then shifted to 1661.25 cm^−1^ after 3 min and further to 1656.96 cm^−1^ after 6 min. Alongside these shifts, the intensity slightly increased after 3 min and decreased after 6 min. These variations suggest that plasma treatment influenced the carbonyl groups in the biopolymer blend more significantly than in gelatin-only samples, indicating structural modifications due to the presence of mucilage and plasma exposure.

The peak associated with the amide III band (∼1175–∼1310 cm^−1^), related to C-N stretching and N-H bending vibrations (Stani et al., 2020), showed shifts for the pH 7 blank aerogels. Initially, the peak at 1249.86 cm^−1^ for 0 min of plasma exposure shifted to 1264.15 cm^−1^ after 3 min and to 1251.29 cm^−1^ after 6 min. These shifts suggest that plasma treatment-induced alterations in the amide III band, likely affecting the C-N and N-H bonding environments in the gelatin matrix.

For the mucilage-gelatin 1:1 aerogel, the peak at 1262.72 cm^−1^ for 0 min shifted slightly to 1264.15 cm^−1^ after 3 min and to 1271.29 cm^−1^ after 6 min. The more pronounced shift in the peak indicates that plasma treatment had a stronger effect on the amide III band in the biopolymer blend, suggesting mucilage influences the response of C-N and N-H bonds to plasma exposure.

The C-O stretching vibration peak (Hanifah et al., 2015) for the pH 7 blank aerogels, observed at 1047.03 cm^−1^ for 0 min of plasma exposure, shifted to 1041.31 cm^−1^ after 3 min and to 1038.46 cm^−1^ after 6 min. This systematic shift to lower wavenumbers suggests that plasma treatment induced modifications in the C-O bonds within the gelatin matrix. These changes could be attributed to plasma-induced cleavage of ester or ether linkages, partial oxidation, or the formation of new hydrogen-bonding interactions. The gradual transition indicates a cumulative effect, where extended plasma exposure results in a more significant alteration of the local bonding environment (Liu et al., 2017). In contrast, the MGA peaked at 1057.03 cm^−1^ for 0 min, shifting to 1047.03 cm^−1^ after 3 min and to 1049.88 cm^−1^ after 6 min. The smaller magnitude of the shift in the MGA indicates that the additional biopolymer component may stabilize the C-O groups or alter their reactivity during plasma treatment.

FTIR analysis revealed notable changes in the peak associated with the crystalline nature (500–700 cm^−1^) of mucilage (Punia and Dhull, 2019), despite the XRD results confirming the aerogels' primarily amorphous structure. For pH 7 blank aerogels, the peak at 581.36 cm^−1^ before plasma treatment (0 min) shifted to 611.36 cm^−1^ after 3 min and to 599.93 cm^−1^ after 6 min. These shifts suggest local structural modifications within the gelatin matrix, consistent with XRD findings showing stable overall crystallinity and minor lattice spacing changes (Fadlelmoula et al., 2022). For the mucilage-gelatin 1:1 aerogels, the peak at 612.79 cm^−1^ at 0 min shifted to 577.08 cm^−1^ after 3 min and to 571.37 cm^−1^ after 6 min. These pronounced shifts, coupled with XRD results confirming an amorphous structure, indicate local rearrangements or interactions between mucilage and plasma. The continuous decrease in wavenumber for MGA suggests that plasma treatment significantly influenced the blend's structural interactions and bonding environments.

Overall, the combined analysis of FTIR and XRD indicates that plasma treatment induces local structural modifications within the aerogels while preserving their fundamental amorphous nature.

Recent studies have explored plasma treatment effects on biopolymers, particularly through FT-IR analysis. Chen et al. (2023) found that air plasma disrupted starch's crystalline structure, decreasing absorption peak intensities at 1 min, with partial recovery at 2–4 min and further depolymerization at 5 min. Low-pressure plasma weakened key peaks (e.g., O-H at 3404 cm^−1^), indicating hydrogen bond interference and increased reactivity (Chen et al., 2023). Similarly, Ledari et al. (2020) showed argon plasma enhanced amide A peaks in gelatin films, increasing surface polar groups and α-helical structures, while bulk hydrocarbon chains remained unchanged (Ledari et al., 2020). These results align with our findings on cold plasma's complex effects on biopolymer structures.

### 3.9 Thermal behavior of aerogels

**Fig. 8:**
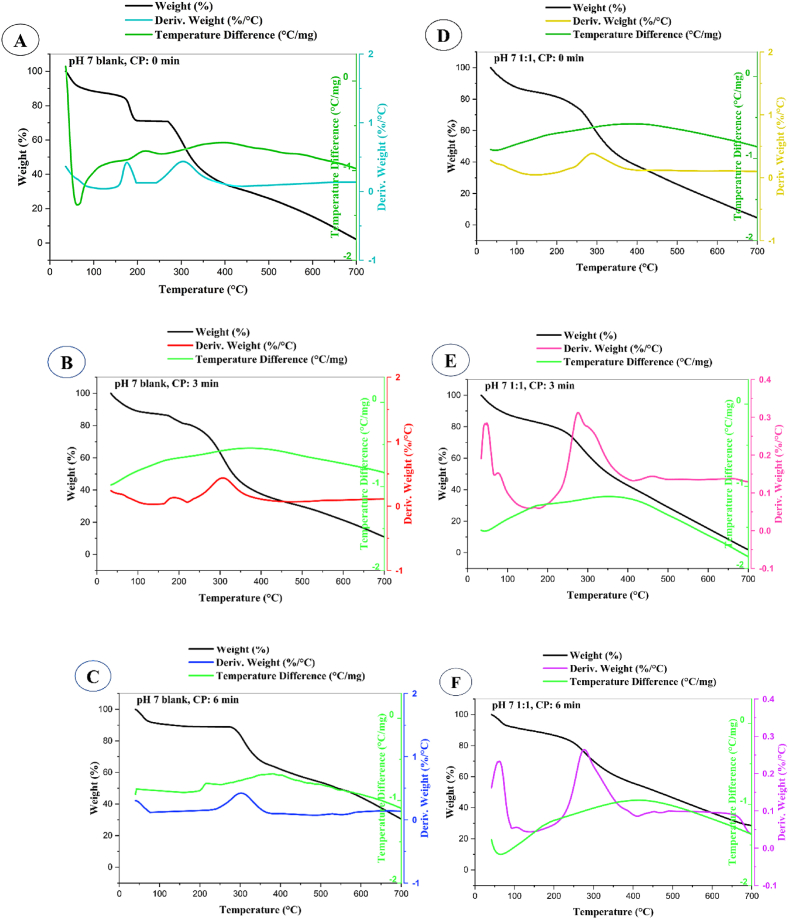
Thermal behavior analysis of aerogels. A, B, and C display the thermogravimetric analysis (TGA), derivative thermogravimetry (DTG), and differential thermal analysis (DTA) results for pH 7 blank aerogels (gelatin only) under various cold plasma treatment times. D, E, and F show the corresponding TGA, DTG, and DTA results for mucilage-gelatin 1:1 aerogels (pH 7 1:1), also under varying cold plasma (CP) treatment durations (0, 3, and 6 min).

Thermogravimetric Analysis (TGA) curves (Fig. 8) illustrating weight (%) versus temperature (°C) were used to assess the thermal stability of gelatin-only (control) and mucilage-gelatin aerogels (MGA; 1:1 ratio, pH 7) under cold plasma (CP) exposure times of 0, 3, and 6 min. Gelatin-only samples without CP treatment (0 min) exhibited significant weight loss (98.93%), which decreased to 90.04% after 3 min of CP, indicating enhanced thermal stability. After 6 min of CP, weight loss further declined to 70.48%, attributed to cross-linking and structural modifications by reactive plasma species (Olatunde et al., 2023). FTIR analysis supports these findings, showing shifts in N-H and C-H stretching vibrations, indicative of chemical modifications enhancing thermal stability.

For MGA, untreated samples (0 min) showed a weight loss of 96.15%, which increased to 99.16% after 3 min of CP, likely due to the disruption of mucilage's stabilizing effect, as correlated with the XRD data (Section 3.6). XRD revealed a peak shift from 13.59° to 21.48° with increased FWHM values, reflecting higher structural disorder and reduced thermal stability. However, after 6 min of CP, weight loss decreased significantly to 71.66%. This aligns with the increased contact angle at 6 min (Section 3.4), suggesting enhanced hydrophobicity due to plasma-induced cross-linking and structural changes. These modifications improve the polymer network's structural integrity, thereby increasing thermal resistance (Sunooj, 2024).

The derivative thermogravimetry (DTG) analysis of gelatin-only aerogels (blank) under various cold plasma (CP) exposure times is presented in Fig. 8. Untreated samples (0 min CP) exhibited major decomposition peaks at 175.93 °C and 304.02 °C with a weight loss rate of 0.4%/°C, indicating significant thermal degradation. After 3 min of CP treatment, these peaks shifted to 188.29 °C (0.13%/°C) and 306.69 °C (0.44%/°C), reflecting improved thermal stability at lower temperatures, although the higher-temperature peak displayed an increased weight loss rate. Prolonged plasma exposure (6 min) resulted in peak shifts to 302.47 °C and 529.15 °C, with reduced rates of 0.42%/°C and 0.09%/°C, respectively, signifying enhanced thermal stability. These results align with the TGA data, confirming structural modifications that reduce the rate of weight loss and increase degradation onset temperatures.

For mucilage-gelatin aerogels (MGA), untreated samples (0 min CP) displayed decomposition peaks at 288.99 °C, 450.27 °C, and 624.35 °C, with weight loss rates of 0.37, 0.12, and 0.10%/°C, respectively. After 3 min of CP treatment, new peaks emerged at 48.45 °C, 75.77 °C, 275.92 °C, and 459.74 °C, with rates of 0.28, 0.15, 0.31, and 0.14%/°C. The reduction in lower-temperature peak stability and rate variations suggest initial structural disruption. At 6 min CP, peaks were observed at 61.76 °C, 104.23 °C, 276.67 °C, and 454.38 °C, with rates of 0.23, 0.05, 0.26, and 0.09%/°C, indicating partial recovery of thermal stability at higher temperatures. This recovery, alongside decreased weight loss rates, supports the TGA findings, suggesting that prolonged CP treatment enhances the thermal stability of MGA by modifying its structural integrity.

Similarly, Alonso-Montemayor et al. (2020) reported that low-pressure rotatory air plasma and atmospheric-pressure air plasma jet treatments markedly enhanced the thermal stability of commercial bleached hemp fibers. Thermogravimetric analysis (TGA) revealed an increase in the initial degradation temperature by 15 °C for fibers treated with low-pressure rotatory air plasma for 30 min, and by 30 °C for fibers subjected to atmospheric-pressure air plasma jet treatment, relative to untreated counterparts (Alonso-Montemayor et al., 2020). In contrast, Chang et al. (2020) noted elevated weight loss rates in starch nanoparticles following oxygen cold plasma treatment, suggesting structural degradation attributed to oxidative effects induced by the plasma process (Chang et al., 2020).

The differential thermal analysis (DTA) was performed on gelatin-only (blank) and mucilage-gelatin 1:1 aerogels to assess further their thermal behavior under various cold plasma exposure times (Fig. 8). For the gelatin-only aerogels with no plasma treatment (0 min CP), DTA identified two significant thermal events. The first peak at 184.43 °C was downward, indicating an endothermic reaction where heat was absorbed. This was consistent with the considerable weight loss observed in TGA and the major decomposition events noted at 175.93 °C and 304.02 °C in DTG. The heat flow rate of 0.24 °C.min/mg reflects the rate of temperature change during the analysis. The second peak at 215.08 °C was upward, indicating an exothermic reaction with heat release, which aligns with the secondary degradation observed in TGA and DTG.

For the gelatin-only aerogels subjected to 6 min of plasma exposure, DTA revealed a first endothermic peak at 202.91 °C and a second exothermic peak at 215.38 °C, corresponding with the improved thermal stability shown in the TGA (with a reduced weight loss of 70.48%) and the DTG data (peaks at 302.47 °C and 529.15 °C with reduced rates of weight loss). The endothermic peak at 202.91 °C correlates with enhanced stability at lower temperatures, while the exothermic peak at 215.38 °C supports the observed improved resistance to thermal degradation. Notably, four out of six samples did not display detectable peaks in the DTA analysis. This absence of peaks suggests that the thermal transitions might have been too subtle or occurred outside the detection range of the DTA measurements. This is similar to observations reported by other research, where high heating rates led to the absence of distinct DTA peaks corresponding to phase transitions due to rapid temperature changes masking subtle thermal events (Kerli et al., 2013). Their study also noted that the presence of hydroxyl groups and impurities in the samples can affect the detection of phase transitions in DTA (Kerli et al., 2013). This highlights the importance of using multiple analytical techniques to capture and understand the samples' thermal behavior fully. Chang et al. (2019) investigated the thermal behavior of starch nanoparticles using thermogravimetric analysis (TGA), revealing three distinct mass loss steps: water evaporation (61.8–67.3 °C), major degradation (294.8–317.6 °C), and further degradation beyond 317.6 °C. Their study found that Waxy Corn Starch and its nanoparticle variants exhibited similar maximum degradation temperatures around 316.3 °C, indicating stable thermal behavior. In contrast, Potato Starch and its nanoparticle counterparts demonstrated lower maximum degradation temperatures (294.8–304.9 °C), suggesting reduced thermal stability due to cold plasma (CP) and ultrasonic treatments. Differential thermal analysis (DTA) supported these results by aligning thermal events with the mass loss steps observed in TGA (Chang et al., 2019).

The combined results from TGA, DTG, and DTA provide a comprehensive understanding of the aerogels' thermal properties and stability, demonstrating that cold plasma treatment enhances thermal resistance through structural modifications. The alignment of DTA with TGA and DTG results underscores the effectiveness of plasma treatment in improving thermal stability, as evidenced by shifts in decomposition temperatures and changes in thermal reaction behavior.

## 4 Conclusions

This study investigated the impact of nitrogen cold plasma (CP) treatment on marshmallow root mucilage-gelatin aerogels (MGA) with varying exposure times (0, 3, and 6 min). This research is groundbreaking in its use of CP treatment for mucilage-gelatin aerogels, highlighting the substantial potential for material customization. The findings revealed significant thermal stability and structural characteristics improvements by employing CP treatment. The research discovered that exposure to CP treatment displayed a shift from increased hydrophilicity at 3 min to improved hydrophobicity at 6 min. Additionally, structural modifications were confirmed through X-ray Diffraction (XRD) and Fourier-transform Infrared Spectroscopy (FTIR) analyses. However, the study's limitations include using only nitrogen plasma and a restricted range of exposure times, as well as not considering the effects of environmental conditions like humidity and temperature. This suggests that future research should explore different plasma gases, varying exposure times, and power levels to fully understand their impact on aerogel properties. Additionally, examining the influence of environmental factors on aerogel stability and performance over time would provide valuable insights.

From an industrial perspective, the findings of this study offer a foundation for scaling up cold plasma-treated biopolymer aerogels for various applications. Given their improved surface properties, these aerogels hold significant potential for large-scale production in sectors such as biomedical engineering, water purification, and food packaging. Future research should focus on optimizing the cold plasma process for cost-effective, high-throughput production while maintaining desirable aerogel characteristics. Overall, this research underscores cold plasma treatment as a promising and scalable technique for enhancing biopolymer-based aerogels, paving the way for their broader adoption in commercial and industrial applications.

## CRediT authorship contribution statement

**Marzieh Rownaghi:** Writing – original draft, Investigation, Formal analysis, Data curation. **Mahdi Keramat-Jahromi:** Writing – review & editing. **Mohammad-Taghi Golmakani:** Writing – review & editing. **Mehrdad Niakousari:** Writing – review & editing, Supervision, Funding acquisition, Conceptualization.

## Declaration of competing Interest

The authors declare that they have no known competing financial interests or personal relationships that could have appeared to influence the work reported in this paper.

## Data Availability

Data will be made available on request.

## Glossary

MGA: Mucilage-gelatin aerogel in a 1:1 (w/w) ratio
CP: Cold plasma
pH 7/pH 5 1:1 Hydrogel/aerogel: Hydrogel/aerogel composed of mucilage and gelatin in a 1:1 (w/w) ratio at pH 7/ pH 5
pH 7/pH 5 blank Hydrogel/aerogel: Hydrogel/aerogel composed of gelatin only at pH 7 / pH 5
FTIR: Fourier-Transform Infrared Spectroscopy
XRD: X-Ray Diffraction
DTA: Differential Thermal Analysis
TGA: Thermogravimetric Analysis
DTG: Derivative Thermogravimetry
BET: Brunauer-Emmett-Teller
BJH: Barrett-Joyner-Halenda
FWHM: Full Width at Half Maximum
